# Lymphadenectomy in women with endometrial cancer: aspiration and reality from a radiation oncologist’s point of view

**DOI:** 10.1186/s13014-015-0460-2

**Published:** 2015-07-16

**Authors:** Robert Foerster, Robert Kluck, Nathalie Arians, Stefan Rieken, Harald Rief, Sebastian Adeberg, Tilman Bostel, Ingmar Schlampp, Juergen Debus, Katja Lindel

**Affiliations:** Department of Radiation Oncology, University Hospital Heidelberg, Im Neuenheimer Feld 400, 69120 Heidelberg, Germany

**Keywords:** Adjuvant radiotherapy, Endometrial cancer, Lymphadenectomy, Prognostic factors, Survival

## Abstract

**Background:**

To investigate the meaning of lymphadenectomy (LNE) in women with endometrial cancer (EC) for clinical outcome and secondly to determine the impact of the method of adjuvant radiotherapy (RT) on survival as well as to define prognostic factors.

**Methods:**

322 patients (pts) underwent adjuvant RT for endometrioid EC at our department from 2004 until 2012 and were included in this retrospective study. Chi-square test, LogRank test and Cox regression were used for statistical analyses.

**Results:**

Median age at diagnosis: 66 years. FIGO stages: FIGO I 69.4 %, FIGO II 15.3 %, FIGO III 14.5 %, FIGO IV 0.9 %. Surgical staging: 30.6 % pelvic/paraaortic LNE, 45 % sole pelvic LNE, 8.8 % sampling of suspicious lymph nodes, 15.6 % no LNE. Adjuvant chemotherapy (ChT): 3.2 %. Sole intravaginal brachytherapy (IVB): 60.2 %. IVB + external beam radiotherapy (EBRT): 39.8 %. 5-year local recurrence free survival (LRFS): 90.6 %, distant metastases free survival (DMFS): 89.8 %, overall survival (OS):79.3 %. In multivariate analysis age (*p* = .007), pT stage (*p* = .029), lymph node status (*p* = .003), grading (*p* = .011) and lymphovascular space invasion (LVSI; *p* = .008) remained as independent prognostic factors for OS. Resection status (*p* = .01) and LVSI (*p* = .014) were independent prognostic factors for LRFS and LVSI (*p* = .008) was the only independent prognostic factor for DMFS. There was no statistically significant survival benefit from LNE in LRFS (*p* = .561), DMFS (*p* = .981) or OS (*p* = .791). 5-year LRFS in stage I and II: 96.0 and 82.9 % after sole IVB, 90.8 and 81.6 % after combined IVB/EBRT (*p* = .105; *p* = .970). 5-year OS rates for stage I and II: 86.5 and 71.3 % after sole IVB, 84.2 % and 69.2 % after combined IVB/EBRT (*p* = .153; *p* = .619).

**Conclusion:**

Comprehensive surgical staging is rarely performed and may be omitted in women with endometrioid EC in stages I-II. Sole IVB delivers equally good local control as combined IVB/EBRT in pts with FIGO stage I and II disease. LVSI deserves more attention as a prognostic factor and these pts may require a combined local and systemic therapy.

## Background

Endometrial cancer (EC) is the most common gynecologic malignancy. While therapy guidelines are widely established and the prognosis is generally favorable, optimal treatment remains controversial. In particular the conductance of systematic lymphadenectomy (LNE) and the role of external beam radiotherapy (EBRT) in early stages have been actively disputed, since both therapy modalities are known to cause substantial morbidity. Current guidelines recommend systematic pelvic and paraaortic LNE with investigation of at least 15 pelvic and 10 paraaortic lymph nodes from FIGO IB onward [1, 2]. However, clinical practice differs among surgical centers and many patients are spared LNE or only undergo sampling of suspicious pelvic lymph nodes commonly omitting paraaortic lymph nodes. For radiation oncologists it can be challenging to recommend the appropriate adjuvant therapy for these patients (pts), especially when they present with additional risk factors, such as grade-3- histology or lymphovascular space invasion (LVSI). We therefore designed this retrospective analysis to elucidate the clinical outcome in the pts treated with adjuvant radiotherapy (RT) at our department and further to investigate the role of LNE and known prognostic factors.

## Methods

Between 2004 and 2012 we performed adjuvant RT in 322 women with endometrioid EC at our department. All pts were included in this retrospective study, which was approved by the ethics committee of the University of Heidelberg. Due to its retrospective and blinded design consent was not required. By revision of the electronic patient charts we collected detailed information on stage, grading, resection status, LVSI, primary surgical therapy, adjuvant RT and additional adjuvant chemotherapy (ChT). FIGO 2009 classification was used for staging and patients were reclassified if necessary. Survival analysis was done for local recurrence free survival (LRFS), distant metastases free survival (DMFS) and overall survival (OS). LRFS was considered to be the time between first diagnosis and first recurrence within the irradiation field. DMFS was calculated as the time from first diagnosis until distant relapse. OS was calculated from date of first diagnosis until death from any cause. Survival was plotted according to Kaplan and Meier. The Log-rank test was used for univariate analysis and Cox proportional hazard model was used for multivariate analysis. The Chi-square test was used to illustrate heterogeneity among treatment groups. A p-value ≤ .05 was considered statistically significant. Statistical analysis was performed with SPSS 22.0 for Windows.

## Results

### Patients’ and tumor characteristics

Pts were first diagnosed with EC at a median age of 66 years (range: 36–92). FIGO stages were distributed as follows: stage I 69.4 %, stage II 15.3 %, stage III 14.5 %, stage IV 0.9 %. Positive lymph nodes (N1) were found in 9.7 %, 24.2 % had an undifferentiated tumor grading (G3) and 16.5 % showed LVSI. Resection was incomplete (R2) in 0.3 %, microscopically positive resection margins (R1) were found in 2.2 % and resection status was indeterminable (Rx) in 5.0 % (Table 1).

**Table 1:** Patients’ and tumor characteristics

Age *N* = 322		
median	66 years	
range	36 − 92 years	
	*n*	%
FIGO *N* = 320		
IA	104	32.5 %
IB	118	36.9 %
II	49	15.3 %
IIIA	13	4.1 %
IIIB	4	1.3 %
IIIC	29	9.1 %
IVA	2	0.6 %
IVB	1	0.3 %
pT stage *N* = 320		
T1	237	74.1 %
T2	58	18.1 %
T3	24	7.5 %
T4	1	0.3 %
Nodal status *N* = 320		
N0	289	90.3 %
N1	31	9.7 %
Grading *N* = 322		
G1	69	21.4 %
G2	175	54.3 %
G3	78	24.2 %
LVSI *N* = 322		
no LVSI	269	83.5 %
LVSI	53	16.5 %
Resection *N* = 322		
R0	298	92.5 %
R1	7	2.2 %
R2	1	0.3 %
RX	16	5.0 %
Metastases N = 320		
M0	319	99.7 %
M1	1	0.3 %

Primary surgical therapy consisted of hysterectomy and bilateral salpingo-oophorectomy in all patients. Pelvic and paraaortic LNE was conducted in 98 pts (30.6 %), 144 (45.0 %) received sole pelvic LNE, 28 (8.8 %) only underwent sampling of suspicious lymph nodes and in 50 (15.6 %) LNE was omitted. RT consisted of EBRT in 1.8 − 2.0 Gy fractions to a cumulative dose of 40.0 − 54.0 Gy and / or high dose rate (HDR) intravaginal brachytherapy (IVB) in 5.0 − 5.5 Gy fractions to a cumulative dose of 10.0 − 22.0 Gy. One hundred twenty eight (39.8 %) pts received combined IVB/EBRT and 194 (60.2 %) received IVB alone. In the IVB/EBRT group the median total EBRT dose was 45.0 Gy and the median total IVB dose was 10.0 Gy. Patients in the sole IVB group received a median total dose of 22.0 Gy. Additional adjuvant ChT consisting of 6 cycles of carboplatin and paclitaxel was given to 3.1 % (*n* = 10) before or after adjuvant RT. Only patients with stage III and IV disease received ChT. A detailed overview on surgical lymph node staging and radiotherapy for FIGO stages I-IV as well as for FIGO stage I according to risk stratification [3] is given in Table 2. Patients with intermediate risk were statistically significantly more often treated with combined EBRT/IVB when surgical lymph node staging was omitted (*p* = .009, Table 2).

**Table 2:** Treatment according to (A) FIGO stages and (B) risk stratification

(A) FIGO stages		*n*	%	*χ* ^2^
FIGO I (*N* = 222)				
LNE	IVB	143	76.9 %	*p* = .194
	IVB + EBRT	43	23.1 %	
No LNE	IVB	24	66.7 %	
	IVB + EBRT	12	33.3 %	
FIGO II (*N* = 49)				
LNE	IVB	21	51.2 %	*p* = .478
	IVB + EBRT	20	48.8 %	
No LNE	IVB	3	37.5 %	
	IVB + EBRT	5	62.5 %	
FIGO III (*N* = 46)				
LNE	IVB	3	7.2 %	*p* = .580
	IVB + EBRT	39	92.8 %	
No LNE	IVB	0	0 %	
	IVB + EBRT	4	100 %	
FIGO IV (*N* = 3)				
LNE	IVB	0	0 %	NA
	IVB + EBRT	2	100 %	
No LNE	IVB	0	0 %	
	IVB + EBRT	1	100 %	
(B) Risk stratification		n	%	*χ* ^2^
Low risk (*N* = 77)				
LNE	IVB	55	94.8 %	*p* = .988
	IVB + EBRT	3	5.2 %	
No LNE	IVB	18	94.7 %	
	IVB + EBRT	1	5.3 %	
Intermediate risk (*N* = 121)				
LNE	IVB	74	70.5 %	*p* = .009
	IVB + EBRT	31	29.5 %	
No LNE	IVB	6	37.5 %	
	IVB + EBRT	10	62.5 %	
High risk (*N* = 24)				
LNE	IVB	14	60.9 %	*p* = .227
	IVB + EBRT	9	39.1 %	
No LNE	IVB	0	0 %	
	IVB + EBRT	1	100 %	

### Survival analysis

**Fig. 1:**
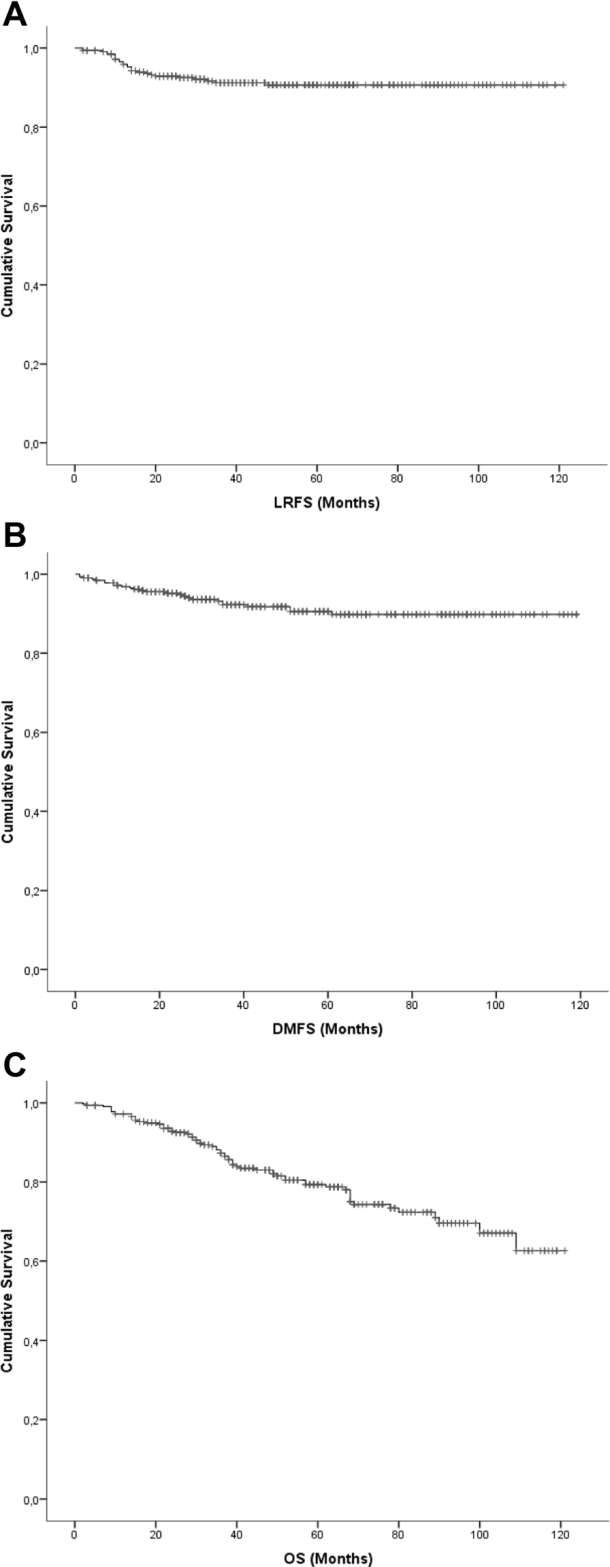
Kaplan-Meier survival curves for **a** LRFS, **b** DMFS, **c** OS

Median follow-up was 49.5 months. Sixty-six (20.5 %) pts died during follow-up, 27 (8.4 %) had a local recurrence and 26 (8.1 %) developed distant metastases. One (0.3 %) patient presented with synchronous distant metastases to the cervical lymph nodes at first diagnosis of endometrioid EC. LRFS, DMFS and OS after 5 years were 90.6, 89.8 and 79.3 % respectively (Fig. 1). Sites of recurrences are shown in Table 3.

**Table 3:** Sites of recurrences

	***n***
Local recurrences *N* = 27	
paraaortic lymph nodes	8
vaginal pole	12
pelvic floor and wall	7
Distant recurrences *N* = 26	
lungs	12
liver	6
skin	3
abdominal wall	2
inguinal lymph nodes	3
cervical lymph nodes	2
retroperitoneal lymph nodes	2
adrenals	2
intestine	3
mediastinum	4
bone	3
peritoneum	4
brain	2

### Univariate analysis

**Fig. 2:**
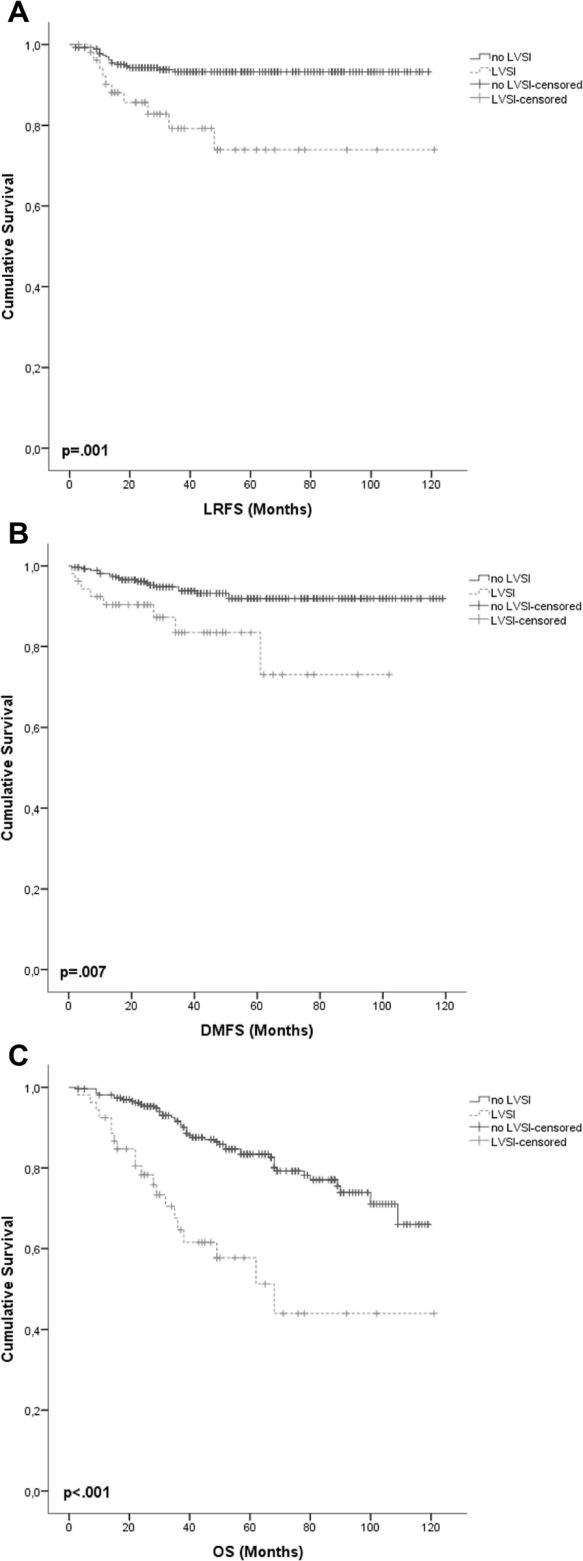
Kaplan-Meier survival curves for LVSI and **a** LRFS, **b** DMFS, **c** OS

Local control was worse with higher pT stage (*p* = .001), positive regional lymph nodes (*p* < .001), positive or indeterminable resection margins (*p* < .001) and LVSI (*p* = .001; Fig. 2)

**Fig. 3:**
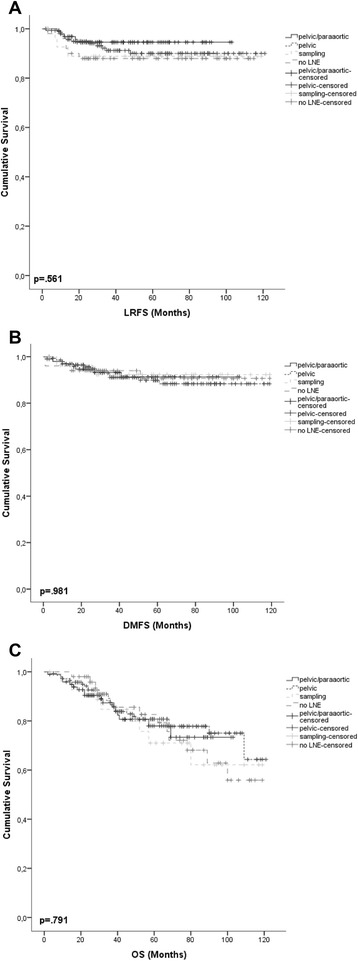
Kaplan-Meier survival curves for LNE and **a** LRFS, **b** DMFS, **c** OS

Distant metastases occurred earlier in pts with higher FIGO stage (*p* = .001), higher pT stage (*p* < .001) and LVSI (*p* = .007; Fig. 2). Age > 66 years (*p* < .045), advanced FIGO stage (*p* < .001), higher pT stage (*p* < .001), positive regional lymph nodes (*p* < .001), higher grading (*p* = .001), positive or indeterminable resection margins (*p* < .001) and LVSI (*p* < .001; Fig. 2) were associated with shorter OS. There was no statistically significant survival benefit from LNE in LRFS, DMFS or OS (Fig. 3). Sole IVB was not inferior to combined IVB/EBRT in early stages independent of the conducted surgical lymph node staging and independent of the risk stratification. Five-year local control in stage I was 96.0 % after sole IVB and 90.8 % after combined IVB/EBRT (*p* = .105). Five-year OS rates in stage I for sole IVB and combined IVB/EBRT were 86.5 % and 84.2 % respectively (*p* = .153). For stage II 5-year LRFS was 82.9 % after sole IVB and 81.6 % after IVB/EBRT (*p* = .970). Five-year OS rates in stage II were 71.3 and 69.2 % for the sole IVB and combined IVB/EBRT groups (*p* = .619). Additional adjuvant ChT did not statistically significantly effect survival in all 3 endpoints.

### Multivariate analysis

Age (*p* = .017; HR 1.88 [95 % CI 1.12 − 3.16]), pT stage (*p* = .029; HR 2.13 [1.08 − 4.21]); lymph node status (*p* = .003; HR 2.74 [95 % CI 1.40 − 5.37]), grading (*p* = .011; HR 1.99 [95 % CI 1.77 − 3.39]) and LVSI (*p* = .008; HR 2.22 [95 % CI 1.24 − 3.98]) were revealed as independent prognostic factors for OS in Cox regression model. LVSI was the only independent prognostic factor (*p* = .008; HR 2.22 [95 % CI 1.24 − 3.98]) for DMFS. Resection status (*p* = .01; HR 3.96 [95 % CI 1.39 − 11.28]) and LVSI (*p* = .014; HR 2.87 [95 % CI 1.25 − 7.10]) remained as independent prognostic factors for LRFS.

### Observed acute toxicities

Women receiving combined IVB/EBRT showed a substantially higher rate of acute gastrointestinal (GI) toxicities compared to those with sole IVB (37.5 % vs. 8.7 %). Only one patient receiving combined IVB/EBRT developed a grade 3 acute GI toxicity (enterocolitis) requiring hospitalization. Regarding acute genitourinary (GU) toxicities we observed similar rates in the IVB/EBRT and sole IVB groups (57.8 % vs. 56.2 %). None developed acute GU toxicities ≥ grade 3.

## Discussion

The conductance of systematic LNE, especially in early stage EC, has been actively disputed over the past years [4]. Two randomized trials did not find a survival benefit from systematic pelvic LNE in EC [5, 6]. Arguable, in both studies paraaortic LNE was omitted, the no-LNE groups showed a high proportion of low-risk pts and adjuvant therapy was not standardized. The Japanese SEPAL trial showed a significantly prolonged OS in intermediate- and high risk patients who underwent combined pelvic and paraaortic LNE, whereas OS was not prolonged in low-risk pts [7]. The uncertainty over the therapeutic value of systematic LNE has led to a point where surgical practice differs among centers substantially and many surgeons prefer an individual risk assessment over the systematic LNE recommended in current guidelines [1, 2]. A recent report from Bogani et al. describes the clinical practice regarding LNE at the Mayo Clinic in Minnesota [8]. The colleagues from Rochester only perform systematic pelvic and paraaortic LNE in pts with >50 % myometrial invasion, non-endometrioid histology or both. Additionally paraaortic LNE is conducted when positive pelvic lymph nodes are found, while pelvic LNE is conducted based on involvement of the uterine cervix, undifferentiated grading and tumor diameter > 2 cm. This practice seems to be supported by a recent Surveillance, Epidemiology, and End Results database analysis from Vargas et al. on risk factors for lymph node metastases in EC [9]. The cohort of women treated with adjuvant RT at our department was also very heterogeneous regarding surgical staging and only a minority underwent comprehensive surgical staging as demanded in current guidelines; however we were unable to detect any statistically significant benefit from surgical lymph node investigation for all 3 endpoints (LRFS, DMFS and OS) in our current study. This was surprising, since we were able to demonstrate a significantly prolonged OS after LNE in pts with type II EC previously [10]. The groups of pts with stage IIIA and IIIB as well as stage IV disease in our current study were too small for LNE- and RT-stratified subgroup analysis (Table 2), but for patients with stage I and II disease we were able to confirm these results in separate analyses. Women with intermediate risk more often received combined IVB/EBRT when LNE was omitted in our study; this, however, did not have a statistically significant influence on survival as well. Our results are generally supported by the findings of the PORTEC trials where no routine LNE was required [11, 12]. We believe that surgical lymph node staging may be omitted in pts with type I EC in stages I-II and we further believe that women who did not receive systematic LNE and are without clinical suspicion of regional lymph node metastases may be treated as if they had undergone comprehensive surgical staging.

We found our results regarding local control, disease-free and overall survival to generally be in line with a recent retrospective study on postoperative RT from Switzerland [13]. Several randomized trials have investigated the role of adjuvant RT in EC [11, 14–16]. All showed a significantly improved local control; even in the treatment of recurrences [17]. Since the Norwegian trial from Aalders et al. [14], published in 1980, efforts have been made to reduce RT-associated morbidity by defining subgroups of pts which do not require EBRT and may benefit from IVB alone [12, 18–22]. The PORTEC-2 trial proved that IVB alone provides similar local control rates as combined IVB/EBRT treatment in pts with higher risk profile in early stages while toxicity is substantially reduced [12, 23]. The women with FIGO stage I and stage II disease in our study cohort also had an equally good local control and OS after sole IVB and combined IVB/EBRT. Far too few patients received additional adjuvant ChT to be able to draw any conclusions on its effect on survival.

In multivariate analysis age over 66 years was associated with a shorter OS (*p* = .017). This is in agreement with a study from Benedetti Panici et al. who reported a reduced overall and cancer specific survival in women over 65 years [24]. Other independent prognostic factors for OS in our analysis were pT stage (*p* = .029), lymph node status (*p* = .003) and grading (*p* = .011). For local control we found resection status (*p* = .01) to be an independent prognostic factor. Interestingly LVSI was the only independent prognostic factor that was found in all 3 endpoints (LRFS [*p* = .014], DMFS [*p* = .049], OS [*p* = .008]) and it was the only independent prognostic factor for DMFS. Previous studies have already reported on the prognostic relevance of LVSI in EC or considered the presence of LVSI a feature of higher risk [13–15, 25–27]. We believe that LVSI deserves more attention as a prognostic factor and that these women may be in need for a combined local and systemic treatment approach.

The observed acute GI toxicities were moderate and in line with a previous report, considering that intensity modulated radiotherapy was available only for patients treated in more recent years [28]. The relatively large proportion (>50 %) of documented acute GU toxicities in both groups is owed to the inclusion of asymptomatic grade 1 vaginal erythema.

## Conclusion

Comprehensive surgical staging is rarely performed and may be omitted in women with type I EC in stages I-II. Sole IVB provides equally good local control as combined IVB/EBRT in stages I and II. Women with LVSI may be in need for a combined local and systemic therapy and LVSI should be included as a major risk factor in future randomized trials.

## Abbreviations

ChT: Chemotherapy
DMFS: Distant metastases free survival
EBRT: External beam radiotherapy
EC: Endometrial cancer
HDR: High-dose rate
IVB: Intravaginal brachytherapy
LNE: Lymphadenectomy
LRFS: Local recurrence free survival
LVSI: Lymphovascular space invasion
OS: Overall survival
pts: Patients
RT: Radiotherapy
